# The effect of an 8-week Life Kinetics exercise program on salivary cortisol, alpha-amylase, and ımmunoglobulin-A in U14 female cross-country skiers

**DOI:** 10.3389/fphys.2026.1847163

**Published:** 2026-07-28

**Authors:** Mehmet Emin Yelken, Burcu Sıla Sezer, Sezgin Hepsert, Hakan Küçüksayan, Nisanur Canikli Temel, Göktuğ Mugan, Umut Yılmaz

**Affiliations:** 1 Ministry of Youth and Sports, Provincial Directorate of Youth and Sports, Cankırı, Türkiye; 2 Faculty of Sports Sciences, Bingol University, Bingöl, Türkiye; 3 Ministry of Education, Elazığ, Türkiye; 4 Department of Medical Biology, Kastamonu University, Kastamonu, Türkiye; 5 Faculty of Sports Sciences, Aydın Adnan Menderes University, Aydın, Türkiye; 6 Independent Researcher, Ankara, Türkiye; 7 Department of Coaching Education, Faculty of Sport Sciences, University of Hakkari, Hakkari, Türkiye

**Keywords:** alpha-amylase and immunoglobulin-A, athletic performance, cortisol, cross-country skiers, salivary

## Abstract

**Introduction:**

The aim of this study is to investigate the effects of an 8-week Life Kinetics exercise program on salivary biomarkers (α-amylase, cortisol, and immunoglobulin-A) in U14 female cross-country skiers.

**Methods:**

A total of 20 female cross-country skiers volunteered to participate in this study. The study was designed as a pre-/post-test controlled experimental study, and adolescent individuals were randomly assigned to either the cross-country skiing (control; *n* = 10) or cross-country skiing + Life Kinetics groups (experimental; *n* = 10). The control group participated in cross-country skiing training for 45 min, 3 days a week, for 8 weeks. The experimental group participated in Life Kinetics exercises in addition to the control group’s exercises. Pre-test and post-test saliva measurements were taken before and after the 8-week protocols.

**Results:**

No significant group, time, or group × time interaction effects were observed for salivary cortisol levels (*p* > 0.05). For α-amylase, a significant group effect was detected (*p* = 0.003), whereas neither the time effect nor the group × time interaction reached statistical significance (*p* > 0.05). For sIgA, the group × time interaction was significant (*p* = 0.003), while the main effects of group and time were not significant (*p* > 0.05). The experimental group demonstrated an increase in sIgA levels from pre- to post-intervention, whereas the sIgA levels decreased in the control group.

**Conclusion:**

The present findings indicate that the 8-week Life Kinetics intervention did not produce significant changes in salivary cortisol levels, suggesting no evidence of altered chronic stress regulation in adolescent female skiers. Although a significant group × time interaction was observed for sIgA, the overall findings should be interpreted cautiously. Taken together, the results suggest that Life Kinetics training was well tolerated from a physiological stress perspective, while further studies with larger sample sizes, longer intervention periods, and repeated biomarker assessments are needed to better clarify its effects on stress- and immune-related responses.

## Introductıon

Adolescence is a critical developmental period characterized by rapid physical, cognitive, and psychosocial changes (Susman and Rogol, 2004; Parlaz et al., 2012; Yaisilp et al., 2025). For adolescent athletes, this period is accompanied by substantial physiological adaptations to training, growth, and competition-related stressors. Consequently, understanding how training interventions influence stress regulation and physiological adaptation during adolescence is of considerable importance (Balla et al., 2024; Janssen and LeBlanc, 2010). In contemporary sport, athletic performance depends not only on physical fitness but also on cognitive and psychological factors, increasing interest in training approaches that simultaneously target motor and mental processes (Yarayan et al., 2025).

Life Kinetics (LK) is a cognitive–motor training approach that combines coordinative movements with cognitive challenges and environmental stimuli (Demirakca et al., 2016; Komarudin, 2019). By promoting the formation of new neural connections, LK exercises are thought to support learning, attention, coordination, and motor adaptation (Dağlı, 2024). Previous studies have reported the beneficial effects of LK interventions in adolescents and athletes from different sport disciplines (Duda, 2015; Agus et al., 2023; Orhan et al., 2021; Tuna et al., 2025; Babazadeh et al., 2025). Recent evidence has also demonstrated that LK exercises may induce favorable cognitive adaptations during puberty, suggesting that this training approach can influence neurocognitive processes during a critical developmental period in adolescents (Sarikabak et al., 2025). However, despite the growing popularity of LK training, its psychophysiological effects remain insufficiently understood.

Stress-related physiological adaptations can be evaluated through salivary biomarkers that reflect neuroendocrine, autonomic, and immune system responses. Cortisol, one of the primary hormones of the hypothalamic–pituitary–adrenal (HPA) axis, is widely used as a marker of chronic stress regulation and training-related physiological load (Tiollier et al., 2005; Erdemir and Tüfekçioğlu, 2008; Hellhammer et al., 2009; Knezevic et al., 2023). Cortisol concentrations may be influenced by pubertal development, exercise load, and psychological stressors in adolescent populations (Groer et al., 2010; Walker et al., 2001; Goetz et al., 2026; Třískala et al., 2026). Salivary α-amylase is considered an indicator of sympathetic–adrenomedullary activity and provides information regarding acute stress responses (Skosnik et al., 2000; Van Stegeren et al., 2006; Schumacher et al., 2013; Barutçu and Yıldız, 2024). In addition, secretory immunoglobulin A (sIgA), the primary antibody in mucosal secretions, plays a central role in immune defense and has been shown to be sensitive to exercise-related stress and recovery status (Gleeson and Pyne, 2000; Deinzer et al., 2000; Otsuki et al., 2004; Takatsuji et al., 2008; Perruzza et al., 2024). These biomarkers together provide complementary information regarding stress regulation and immune adaptation in athletes.

Although the contribution of LK exercises to motor learning and performance development has been increasingly investigated (Duncan et al., 2018; Fauzi et al., 2024), evidence regarding their effects on stress- and immune-related physiological responses remains limited. This gap is particularly evident in adolescent athletes, a population characterized by ongoing biological maturation and heightened sensitivity to training-related stressors. Therefore, this study aimed to investigate the effects of an 8-week LK exercise program on salivary cortisol, α-amylase, and sIgA levels in U14 female cross-country skiers. By examining biomarkers associated with HPA–axis regulation, autonomic nervous system activity, and mucosal immunity, this study provides novel evidence regarding the psychophysiological effects of LK training in adolescent athletes.

## Method

This study was conducted using a pre-test–post-test controlled experimental research design to examine the effect of LK exercise intervention on physiological responses (Thomas et al., 2023). This design is widely preferred in exercise and sports science because it allows for the comparison of time-dependent changes and between-group differences through pre- and post-intervention measurements.

### Calculation of sample size

Sample size estimation was conducted *a priori* using G*Power software (version X.X). Based on a large expected effect size (Cohen’s *d* = 0.80), a significance level of *α* = 0.05, and a statistical power of 80% (1−*β* = 0.80), the analysis indicated that a minimum sample size of 24 participants (12 per group) would be required. To compensate for potential attrition, recruitment was planned accordingly. However, four athletes voluntarily withdrew from the study during the intervention period for personal reasons, resulting in a final sample of 20 participants (10 participants per group). Although the final sample size was lower than initially estimated, participant attrition occurred equally across groups, thereby preserving group balance. Furthermore, the primary analyses were conducted using linear mixed-effects models fitted with restricted maximum likelihood (REML), which are generally considered more efficient than traditional methods for repeated-measures data because they account for within-subject variability and make use of all available observations. The *a priori* power analysis applied when calculating the sample size was performed using the G*Power 3.1.9.7 (Franz Faul, University of Kiel, Germany) program. The 20 athletes participating in the study were randomly assigned to groups. Randomization was performed using the web-based Randomization Software developed by the Department of Biostatistics and Medical Informatics at İnönü University. The randomization sequence was generated and implemented by the third author (Sezgin Hepsert), who was solely responsible for assigning participants to study groups. Only the researcher who conducted the randomization procedure had access to the group assignment information. To minimize potential assessment bias, laboratory personnel responsible for biochemical analyses were not informed of the group assignments throughout the study. Outcome measurements and laboratory analyses were performed independently of the randomization process.

### Research group and ethical approval

The research was conducted in Çankırı province, Turkey, with a total of 20 participants who had been involved in cross-country skiing for 3 years, held a license, and met the inclusion criteria. Written permission was obtained from the Scientific Research and Publication Ethics Committee of Bingöl University Faculty of Health Sciences, dated May 30, 2025—E.213301, to review the scientific and ethical appropriateness of the research. The research was conducted in accordance with the Declaration of Helsinki, and the participants and their families were informed about the purpose, structure, and procedure of the research. Written informed consent forms were also obtained from the participants and their parents.

### Inclusion criteria for the study

This study included individuals aged 12–14 years who were in the U14 age category, had at least 3 years of regular cross-country skiing training experience, and possessed the physical fitness to participate regularly in the planned training and activities during the study period. Additionally, the participants were required to voluntarily agree to participate in the study, sign an informed consent form with parental/guardian approval, and have no history of neurological, psychiatric, or serious orthopedic illness or injury within the past 6 months.

### Exclusion criteria

Individuals who did not participate in two or more applications, who received a psychiatric or psychological diagnosis before or during the study, who did not cooperate sufficiently with saliva sample collection procedures, who experienced a serious health problem during exercise applications, or who were disabled to a degree that prevented them from completing the application were excluded from the study. Furthermore, if a request to withdraw voluntary participation was received from the participant or guardian during the study process, the individual’s data were not included in the analyses.

**Figure 1:**
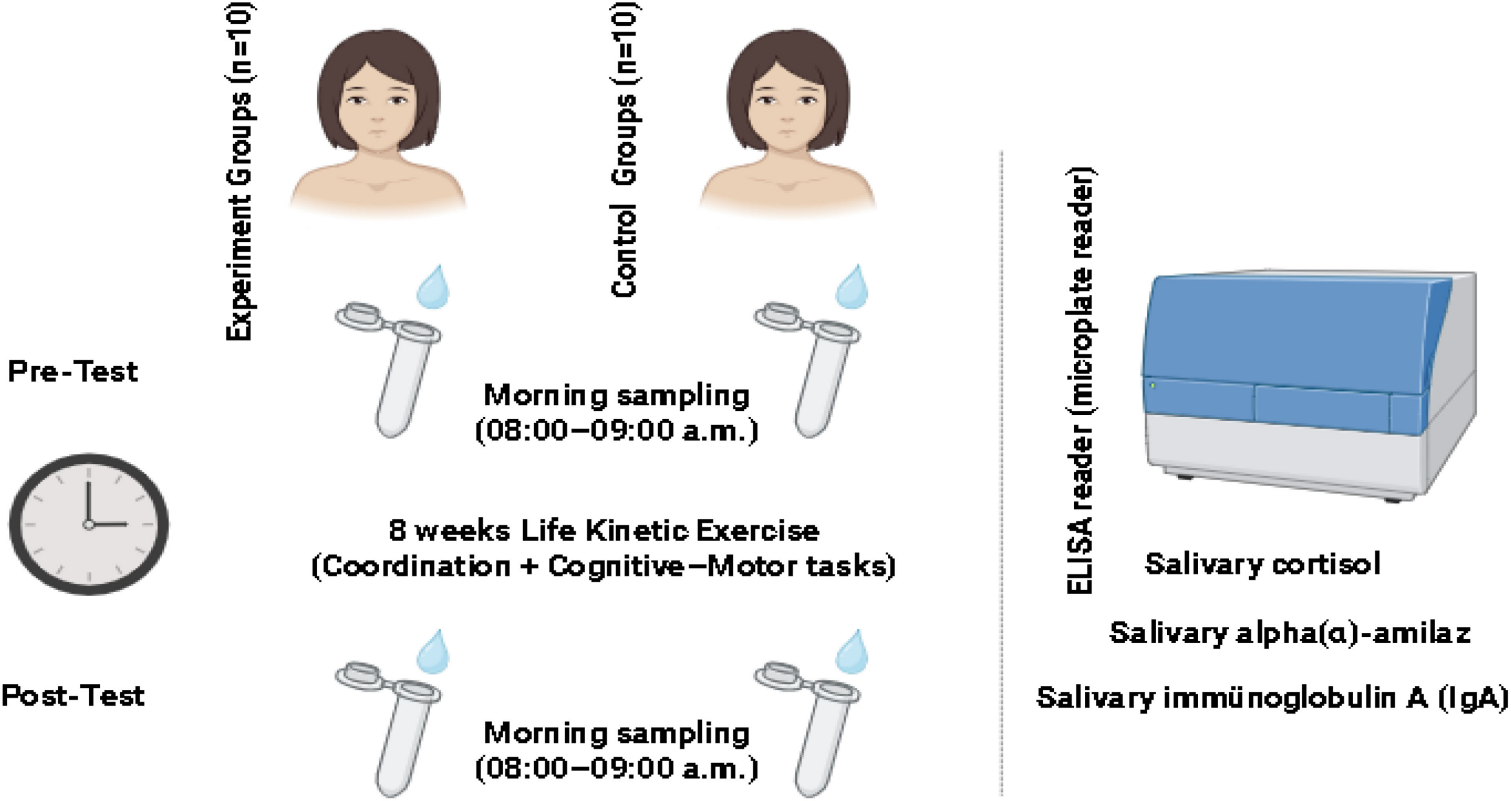
Graphic summary information related to the research.

**Figure 1** shows a pre-/post-test controlled experimental design model used to evaluate the effects of the LK exercise program on physiological stress and immune responses. It is also summarized that saliva samples were collected from the experimental and control groups in the morning (08:00–09:00) before and after the intervention and that the levels of salivary cortisol, α-amylase, and sIgA in the obtained samples were analyzed using the ELISA method.

Based on **Table 1**, the baseline (pre-test) anthropometric characteristics of the experimental group were 12.70 ± 0.30 years for age, 153.10 ± 2.15 cm for height, and 40.71 ± 3.03 kg for body weight. In the control group, age was 13.30 ± 0.39 years, height was 157.20 ± 2.67 cm, and body weight was 45.64 ± 2.39 kg. Between-group comparisons yielded *t* = 4.679 (*p* = 0.002) for age, *t* = 3.796 (*p* = 0.002) for height, and *t* = 4.078 (*p* = 0.007) for body weight.

**Table 1:** Descriptive statistics regarding the participants.

Groups	*n*	Age (year)	Height (cm)	Body weight (kg)
Experimental	10	12.70 ± 0.30	153.10 ± 2.15	40.71 ± 3.03
Control	10	13.30 ± 0.39	157.20 ± 2.67	45.64 ± 2.39
		*p* = 0.002 /*t* = 4.679	*p* = 0.002/*t* = 3.796	*p* = 0.007/*t* = 4.078

### Life Kinetics ıntervention protocol

Within the scope of this study, the LK exercise protocol was applied to 10 cross-country skiers for 8 weeks, three days per week (Monday, Wednesday, and Friday), between 10:00 and 11:00. Saliva samples were collected 30 min prior to the first exercise session. Each training session lasted approximately 45 min. All LK sessions were structured into standardized phases consisting of warm-up, main exercise, and cool-down in accordance with established exercise science and cognitive–motor training literature (Behm and Chaouachi, 2011; Jerath et al., 2006). The entire intervention process was directly supervised by the researcher to ensure implementation fidelity and protocol standardization. The LK program was organized into four progressive 2-week phases (weeks 1–2, 3–4, 5–6, and 7–8). Exercise intensity was not controlled through physiological load indicators (e.g., heart rate) but rather through progressive increases in cognitive–motor task demand. Progression was systematically achieved by increasing task complexity, enhancing dual-task requirements, reducing response time, increasing sensory (visual and auditory) stimulus complexity, and incorporating progressively more challenging unstable-surface conditions. This structure was designed to ensure a gradual and systematic increase in cognitive load throughout the intervention period. The weekly content targeted specific cognitive–motor domains, including motor coordination and visual–cognitive integration (weeks 1–2), working memory and divided attention (weeks 3–4), reaction time and dual-task performance (weeks 5–6), and cognitive flexibility and executive functions (weeks 7–8). Detailed task examples for each phase are presented in **Table 2** (Sarikabak et al., 2025). All participants continued their standard cross-country skiing training prescribed by their club coaches throughout the study period. Training frequency, duration, and sport-specific workload were kept equivalent between experimental and control groups, and no additional training camps, competitions, or supplementary conditioning programs were introduced. Therefore, equivalence in sport-specific training volume was ensured between groups. The experimental group received the LK intervention in addition to standard skiing training, resulting in an additional supervised cognitive–motor training load of approximately 135 min per week (3 × 45 min sessions). Thus, the only systematic difference between groups was the additional LK-related cognitive–motor exposure. Attendance was recorded for all sessions, and 100% adherence was achieved across all participants. Implementation fidelity was ensured through standardized delivery of all sessions by the same researcher using a fixed protocol throughout the intervention period. This experimental design maintained equivalence in sport-specific training load between groups while allowing evaluation of whether observed changes could be attributed to the specific cognitive–motor characteristics of the LK intervention rather than differences in total training exposure.

**Table 2:** Kinetics exercise program.

Week	Main theme	Targeted cognitive–motor components	Type of task performed (each session ≈45 min)
1–2	Basic coordination and visual–cognitive integration	Cross-coordination, visual attention, balance control	Cross-limb coordination walks (eyes open/closed), simultaneous upper limb tasks, visual cue-based guidance, simple cognitive tasks on a balance surface
3–4	Motor–cognitive multitasking applications	Working memory, divided attention, postural control	Jumping and direction-changing tasks, simultaneous counting tasks, sequential motor memory applications, cognitive processing on unstable surfaces
5–6	Reaction rate and dual-task management	Reaction time, decision-making, dual-task capacity	Visual/auditory stimulus-based directional changes, variable ball control tasks, visual attention-based matching, simultaneous motor and verbal tasks
7–8	Mixed cognitive load and mental resilience	Sustained attention, cognitive flexibility, executive functions	Time-limited attention tasks, simultaneous dual-task cognitive tasks, narrative-based tasks on unstable surfaces, sequential decision-making applications

The LK exercise program presented in **Table 2** is structured based on training models targeting cognitive–motor integration, dual-task applications, attention, and executive functions (Woollacott and Shumway-Cook, 2002; Diamond, 2015; Stein et al., 2017; Peker, 2014).

Throughout the study period, all participants continued with the standard cross-country skiing training program planned by the club coaches and appropriate for their age group. The weekly training frequency and duration were kept similar across groups, and no additional loading, camps, or competition periods were planned during the study. Thus, it was intended that any observed differences in physiological and biochemical variables would be attributable to the LK exercise intervention rather than routine cross-country skiing training.

### Anthropometric measurements

Body mass measurements were taken in the morning at 8:00 a.m. on an empty stomach, while barefoot and wearing only shorts, using a scale accurate to ±20 g before the intervention program was implemented in the first week of the study. Height measurements were taken using a sliding caliper while the students stood upright. The scale was adjusted so that the sliding caliper heads touched the subjects, and the length was recorded with an accuracy of ±1 mm (Louer et al., 2017).

### Saliva sampling procedure

Saliva samples were used in the study to evaluate the target biomarkers. Saliva-based assays are widely preferred in human populations due to their non-invasive nature and high acceptability (Granger et al., 2007; Nater and Rohleder, 2009). Saliva samples were collected from all participants under standardized conditions. Prior to sampling, the participants were instructed not to consume food or drink, brush their teeth, or chew gum for at least 30 min. Samples were collected at the same time of day for each measurement to minimize the influence of the circadian rhythm (08:00–09:00) (Granger et al., 2012). All samples were collected under fasting conditions. Saliva collection was performed using the unstimulated drool method, and the participants were asked to provide an adequate volume of saliva into sterile tubes. The participants were instructed to remain seated and avoid excessive oral movements during sample collection. Samples showing oral bleeding or contamination were excluded from the analyses. The collected saliva samples were immediately labelled and stored at –20 °C until the time of analysis. This procedure was carried out in accordance with standards recommended in the literature to preserve the stability of saliva biomarkers and enhance measurement reliability (Gröschl, 2008).

### ELISA analysis procedure

Frozen saliva samples were thawed at room temperature and centrifuged at 1,500 × *g* for 10–15 min prior to analysis. Salivary cortisol, α-amylase, and secretory immunoglobulin A (sIgA) concentrations were determined using commercially available ELISA kits according to the manufacturers’ instructions (YL Biont, Shanghai, China; cortisol: YLA0549HU, α-amylase: YLA0819HU, and sIgA: YLA0762HU). All samples were analyzed in duplicate, and laboratory personnel were blinded to the participants’ group allocation. According to the manufacturer’s specifications, intra-assay coefficients of variation were <8% and inter-assay coefficients of variation were <10% for all assays. The analytical sensitivities and assay ranges were 0.251 and 0.5–100 ng/mL for cortisol, 10.13 and 20–6,000 U/L for α-amylase, and 0.01 and 0.02–7 μg/mL for sIgA, respectively.

The enzyme-linked ımmunosorbent assay (ELISA) method was used for the quantitative determination of cortisol, α-amylase, and secretory sIgA levels in saliva samples. ELISA is a reliable immunological assay method that enables the measurement of biomarkers at low concentrations with high sensitivity and specificity (Lequin, 2005). Prior to analysis, frozen saliva samples were thawed at room temperature and centrifuged at 1,500 × *g* for 10–15 min to remove particulate matter. The supernatant was collected and used for the analyses. Measurements were performed using commercial ELISA kits developed for the relevant biomarkers in accordance with the manufacturer’s instructions (YL Biont, USA; cortisol: YLA0549HU, α-amylase: YLA0819HU, and sIgA: YLA0762HU). All standards, controls, and samples were analyzed in duplicate. Standard curves were constructed using serial dilutions of standard solutions provided by the manufacturer. Following the incubation and washing steps, tetramethylbenzidine (TMB) substrate was added, the reaction was terminated with a stopping solution, and optical density values were measured at a wavelength of 450 nm using a microplate reader. Sample concentrations were calculated using the standard curves obtained. According to the manufacturer’s specifications, all assays demonstrated acceptable analytical precision, with intra-assay coefficients of variation below 8% and inter-assay coefficients of variation below 10%. The analytical sensitivities and assay ranges were 0.251 and 0.5–100 ng/mL for cortisol, 10.13 and 20–6,000 U/L for α-amylase, and 0.01 and 0.02–7 μg/mL for sIgA, respectively. To enhance analytical reliability, all assays were performed under standardized laboratory conditions, and laboratory personnel responsible for ELISA analyses were blinded to the participants’ group allocation. This method has been reported as a valid and reliable approach for assessing stress and immune responses in child and adolescent athletes (Jaedicke et al., 2012).

### Data analysis

The data were analyzed using SPSS 25.0 (IBM, Armonk, NY, USA). Prior to the main analyses, the assumptions underlying the statistical procedures were carefully evaluated. The data distribution was initially assessed using skewness and kurtosis coefficients, and this assessment indicated acceptable normality for all outcome variables. Additionally, histograms, Q–Q plots, and standardized residual distributions were visually examined (George and Mallery, 2019). Potential outliers were examined using standardized residuals, and no significant outliers requiring exclusion were identified. Homogeneity of variance was assessed using plots of residuals versus adjusted values and was found to be satisfactory. Given the repeated measures design and the relatively small sample size, an adapted linear mixed-effects model using the restricted maximum likelihood (REML) estimator was employed instead of traditional repeated measures ANOVA. This approach was preferred because, while it is less sensitive to violations of the sphericity assumption, it provides greater flexibility in modeling within-subject correlations and individual variability over time. The fixed effects included group (experimental and control), time (pre-test and post-test), and their interaction, while participants were included as a random effect. Model adequacy was assessed through a visual examination of residual plots and homoscedasticity plots, and no significant deviations from the model assumptions were found. Where a significant main effect or interaction was detected, Tukey’s multiple comparison test was used. Model assumptions were visually verified using residual distribution and homoscedasticity plots, confirming no significant violations. Effect size was calculated using partial eta-squared (ηp²) and interpreted according to Cohen’s classification (small = 0.01, medium = 0.06, and large ≥ 0.14) (Lakens, 2013). The level of statistical significance was set at *p <*0.05. The graphs were prepared using the GraphPad Prism 8 software.

### Findings

The cortisol levels were examined, and in the experimental group, the mean value was 67.83 ± 10.72 at pre-intervention and 69.77 ± 6.96 at post-intervention. In the control group, the cortisol level was 78.27 ± 39.02 at pre-intervention and 76.92 ± 30.28 at post-intervention (**Table 3**).

**Table 3:** Statistical data for pre-test and post-test measurements of cortisol, α-amylase, and sIgA.

Variables	Measurement times	Mean ± SS
Cortisol	Experimental group (pre)	67.83 ± 10.72
	Experimental group (post)	69.77 ± 6.96
	Control group (pre)	78.27 ± 39.02
	Control group (post)	76.92 ± 30.28
α-Amylase	Experimental group (pre)	2262 ± 257.3
	Experimental group (post)	2654 ± 294.2
	Control group (pre)	2316 ± 751.7
	Control group (post)	2933 ± 570.7
sIgA	Experimental group (pre)	121.3± 12.82
	Experimental group (post)	143 ± 18.13
	Control group (pre)	149.9 ± 56.96
	Control group (post)	105.3 ± 27.24

The α-amylase levels were assessed in both groups at pre- and post-intervention measurements. In the experimental group, the mean value was 2,262 ± 257.3 at pre-test and 2,654 ± 294.2 at post-test. In the control group, the mean α-amylase level was 2,316 ± 751.7 at pre-test and 2,933 ± 570.7 at post-test (**Table 3**).

The sIgA levels were evaluated in both groups at pre- and post-intervention measurements. In the experimental group, the mean value was 121.3 ± 12.82 at pre-test and 143 ± 18.13 at post-test. In the control group, the mean sIgA level was 149.9 ± 56.96 at pre-test and 105.3 ± 27.24 at post-test (**Table 3**).

Upon examining the results of the analysis of cortisol levels, the group effect (*F*(1, 36) = 0.001, *p* = 0.971, ηp² = 0.003), the time effect (*F*(1, 36) = 1.189, *p* = 0.282, ηp² = 0.032), and the group × time interaction (*F*(1, 36) = 0.041, *p* = 0.839, ηp² = 0.001) were found to be statistically insignificant (**Table 4**).

**Table 4:** Analysis results for the variables cortisol, α-amylase, and sIgA.

Variable	Effect	*F* (1, 36)	95% CI	ηp²	*p*
Group	0.001	-16.65 to 16.07	0.003	0.971
Cortisol	Time	1.189	-7.564 to 25.16	0.032	0.282
	Group × time	0.041	-36.00 to 29.44	0.001	0.839
	Group	9.768	-832.4 to -177.2	0.213	0.003
α-Amylase	Time	1.064	-161.0 to 494.2	0.029	0.309
	Group × time	0.483	-430.6 to 879.7	0.013	0.491
	Group	1.176	-10.03 to 32.89	0.031	0.287
sIgA	Time	0.182	-25.98 to 16.94	0.005	0.672
	Group × time	9.794	-109.2 to -23.31	0.214	0.003

When the analysis results regarding the α-amylase levels were examined, it was found that the group effect was statistically significant (*F*(1, 36) = 9.768, *p* = 0.003, ηp² = 0.213), while the time effect (*F*(1, 36) = 1.064, *p* = 0.309, ηp² = 0.029) and the group × time interaction (*F*(1, 36) = 0.483, *p* = 0.491, ηp² = 0.013) were found to be statistically insignificant (**Table 4**).

When the analysis results regarding sIgA levels were examined, it was found that the group effect (*F*(1, 36) = 1.176, *p* = 0.287, ηp² = 0.031) and the time effect (*F*(1, 36) = 0.182, *p* = 0.672, ηp² = 0.005) were not statistically significant; however, the group × time interaction was found to be statistically significant (*F*(1, 36) = 9.794, *p* = 0.003, ηp² = 0.214) (**Table 4**).

## Discussion

LK exercises, which have the potential to support an individual’s cognitive, motor, and psychomotor functions through a holistic approach, have become a notable area of application among different training and exercise approaches in recent years. With their multi-task-based structure, LK applications aim to simultaneously stimulate the central nervous system, triggering the brain’s biochemical and functional adaptation processes through external stimuli and supporting the formation of new learning mechanisms. In this context, the present study aimed to investigate the effects of regular LK exercise intervention on stress response and immune system-related biomarkers in female cross-country skiers, thereby determining whether this intervention approach is safe and sustainable in terms of physiological adaptation and responses to exercise load.

This study demonstrates that an LK exercise intervention administered to female cross-country skiers over an 8-week period did not result in a statistically significant change in cortisol levels obtained from saliva samples in pre- and post-test measurements. Similarly, comparisons between the control group and the experimental group revealed no significant difference in post-test cortisol levels. This finding indicates that the training protocol did not induce a chronic or excessive stress response in the hypothalamic–pituitary–adrenal (HPA) axis. The literature indicates that moderate-intensity training involving cognitive–motor integration or technique-focused training has limited effects on cortisol response in adolescent athletes (Cadegiani and Kater, 2017; Knatauskaitė et al., 2021). It has been reported that the cortisol levels remain stable or show only acute and transient changes in exercise models that involve cognitive load but do not create high metabolic stress (Meeusen et al., 2013; Halson, 2014; Budde et al., 2025). However, high-volume training periods and inadequate recovery may lead to elevated cortisol concentrations in young athletes (Rohleder et al., 2007; Papacosta and Nassis, 2011; Smithberg, 2018). From a chronic stress perspective, the absence of significant changes in cortisol may indicate that the LK intervention did not impose a physiological load sufficient to disrupt HPA axis regulation in adolescent athletes. Since salivary cortisol is widely accepted as a marker of chronic stress adaptation and endocrine regulation, the present findings suggest that the additional LK training was well tolerated and did not contribute to excessive physiological stress. Furthermore, systematic data suggest that appropriately prescribed exercise may contribute to maintaining cortisol homeostasis, particularly in youth populations (Hanke et al., 2023; Li et al., 2025). This interpretation is further supported by evidence indicating that adolescent athletes exhibit a relatively adaptive and protective HPA axis profile, which may help maintain hormonal stability in response to moderate training stimuli (Gunnar and Quevedo, 2007; DeJoseph et al., 2024). In addition, Ciaccioni et al. (2024) emphasized that salivary cortisol is an important biomarker of sport-related stress and highlighted that cortisol responses should be interpreted within the broader physiological and psychological demands of the sporting environment. Their findings also suggest that repeated saliva sampling across multiple time points may provide a more comprehensive assessment of stress-related endocrine regulation than isolated pre- and post-intervention measurements. Therefore, the absence of significant cortisol alterations in the present study should be interpreted within the methodological context of the sampling protocol employed. Taken together, the present findings suggest that the 8-week LK intervention was well tolerated from an endocrine perspective and did not appear to induce chronic-stress-related alterations in cortisol regulation among adolescent female athletes.

In the present study, a statistically significant group effect was observed for salivary α-amylase levels, whereas neither the main effect of time nor the group × time interaction reached statistical significance. Furthermore, comparisons between the groups revealed significant differences in α-amylase levels independent of temporal changes. Salivary α-amylase is considered a sensitive indicator of sympathetic–adrenomedullary (SAM) system activity and is commonly used as a marker of acute stress responses associated with cognitive, attentional, and environmental demands (Nater and Rohleder, 2009). The literature reports that exercise models involving coordination and cognitive processing may influence α-amylase activity (Strahler et al., 2010; Engert et al., 2011), while increased attentional demands have also been associated with elevated α-amylase responses (Akizuki and Ohashi, 2014; Asakura, 2024). However, because neither the main effect of time nor the group × time interaction was statistically significant, the present findings do not support a progressive intervention-related change in α-amylase levels. The observed group difference rather appears to reflect differences that were independent of temporal adaptation during the intervention period. Similarly, studies have shown that moderate-intensity exercise programs may not substantially alter α-amylase responses over time (Fakharirad et al., 2020), whereas acute and high-intensity exercise is generally associated with more pronounced elevations (Chatterton et al., 1996; Weiss et al., 2019). Within the broader framework of stress physiology, the stable α-amylase response observed in the present study may indicate that the LK intervention did not induce excessive sympathetic activation despite the additional cognitive–motor demands imposed on the athletes. This interpretation is consistent with the notion that salivary α-amylase reflects acute autonomic responses rather than chronic physiological adaptation. In addition, Ciaccioni et al. (2024) suggested that the combined interpretation of α-amylase and cortisol responses may provide complementary information regarding the interaction between sympathetic activation and HPA axis regulation. Taken together, the α-amylase findings suggest that the LK program did not produce marked alterations in acute-stress-related autonomic activity during the intervention period.

In this study, neither the main effect of group nor the main effect of time reached statistical significance for salivary sIgA levels. However, a significant group × time interaction was observed, indicating that the pattern of change in sIgA levels over time differed between the control and experimental groups. This finding suggests that the intervention may have influenced the temporal pattern of mucosal immune responses rather than producing a uniform overall increase or decrease in sIgA concentrations. A literature review indicates that moderate-intensity, regular, and well-tolerated exercise protocols generally maintain sIgA levels or support immune function (Campbell and Turner, 2018; Walker et al., 2001; Walsh and Oliver, 2016; Walsh, 2018). In this context, the absence of significant main effects of group and time is consistent with the literature suggesting that moderate training loads do not necessarily induce marked alterations in mucosal immunity. Furthermore, the literature reports decreases in sIgA levels and an increased risk of upper respiratory tract infections, particularly during training periods characterized by high volume, high intensity, or inadequate recovery (Nieman and Wentz, 2019; Walsh, 2018). Therefore, the absence of an overall decline in sIgA levels in the present study may indicate that the additional LK training load did not result in immune suppression. The significant group × time interaction observed for the sIgA levels may reflect differential immune adaptations between groups throughout the intervention period rather than an overall effect of group or time alone. It is known that immune parameters in adolescent athletes can be influenced by growth, hormonal variability, sleep patterns, and psychosocial stress factors (Simpson et al., 2015). Accordingly, the interaction effect should be interpreted cautiously and within the broader context of physiological adaptation and individual variability. The literature indicates that cognitive–motor-based exercise approaches may support adaptation without provoking excessive inflammatory or physiological stress (Herold et al., 2020; Stillman et al., 2020). Taken together, the present findings suggest that the LK program may have influenced the trajectory of mucosal immune responses over time while preserving overall immune homeostasis in adolescent female athletes.

### Limitations

The limited sample size and the fact that the participants consisted solely of female cross-country skiers of a similar age group restricted the generalizability of the findings. However, this homogeneous structure supported internal validity. Additionally, statistically significant differences were observed between the experimental and control groups in certain baseline anthropometric characteristics, indicating a degree of heterogeneity at the beginning of the study. Although the primary outcome variables (saliva-based biomarkers) exhibited comparable baseline values between groups, the presence of these anthropometric differences should be acknowledged as a potential limitation when interpreting the findings. Stress and immune responses were assessed solely through saliva-based biomarkers; while this method offers a non-invasive advantage in female athletes, it was not supported by serum or cellular immune markers. Furthermore, the 8-week duration of LK exercise may be considered limited, particularly in terms of revealing the long-term effects of immunological adaptations. In addition, the experimental group participated in supplementary LK training sessions beyond their regular sport-specific training. Therefore, the observed effects cannot be attributed exclusively to the specific cognitive–motor characteristics of LK exercises, as the potential influence of the additional training volume cannot be completely excluded. Finally, the lack of evaluation of physiological responses alongside performance or cognitive outcomes limits a more comprehensive interpretation of the functional effects of LK exercises. Moreover, several potential confounding factors, including maturation status, training history and adaptation level, seasonal variation, recent infection history, menstrual cycle phase, sleep quality, nutritional status, psychological stress, and competition schedule, were not systematically controlled or monitored. Therefore, the potential contribution of individual biological and contextual variability to the observed physiological and immunological responses cannot be excluded and should be considered when interpreting the findings. Taken together, these limitations should be acknowledged when interpreting the present results and considered in the design of future studies.

## Conclusion

The present study investigated the effects of the LK intervention on stress-related physiological responses and immune system markers in adolescent female cross-country skiers. The findings indicated no significant effects of the intervention on salivary cortisol levels, whereas a significant group effect was observed for salivary α-amylase and a significant group × time interaction was observed for sIgA levels. These results suggest that LK exercises may be associated with differences in selected stress- and immune-related biomarkers without inducing marked alterations in cortisol responses.

With respect to α-amylase, the observed group-related differences may indicate variations in physiological responses associated with sympathetic nervous system activity. However, because neither the main effect of time nor the group × time interaction was significant, these findings should be interpreted with caution. When evaluated in terms of immune system parameters, LK interventions do not appear to create negative immune stress in adolescent athletes, and the absence of significant main effects on sIgA levels, together with the observed group × time interaction, suggests that mucosal immune responses were maintained while exhibiting different temporal response patterns between groups. This suggests that LK, which prioritizes cognitive–motor integration and does not involve high metabolic load, may represent a well-tolerated training approach for athletes in their developmental years.

In conclusion, it can be said that LK exercises may influence selected stress- and immune-related biomarkers in young female cross-country skiers without evidence of immune suppression. However, future studies involving larger sample groups, different sports disciplines, and longer-term interventions will contribute to a more comprehensive understanding of the physiological and functional effects of LK.

## Data Availability

The original contributions presented in the study are included in the article/supplementary material. Further inquiries can be directed to the corresponding author.
